# Dolphin whistles can be useful tools in identifying units of conservation

**DOI:** 10.1186/s40850-021-00085-7

**Published:** 2021-07-29

**Authors:** Elena B. Papale, Marta A. Azzolin, Irma Cascão, Alexandre Gannier, Marc O. Lammers, Vidal M. Martin, Julie N. Oswald, Monica Perez-Gil, Rui Prieto, Mónica A. Silva, Marco Torri, Cristina Giacoma

**Affiliations:** 1 Institute for the Study of Anthropic Impacts and Sustainability in the Marine Environment (CNR-IAS), unit of Capo Granitola, National Research Council, Via del Mare 3, 91021 Campobello di Mazara, TP Italy; 2 Life Sciences and Systems Biology Department, University of Torino, Via Accademia Albertina 13, 10123 Torino, Italy; 3 IMAR – Institute of Marine Research & OKEANOS R&D Centre; University of the Azores, Horta, Portugal; 4 Groupe de Recherche sur les Cétacés, Antibes, France; 5 Hawaii Institute of Marine Biology, University of Hawaii, Kaneohe, HI 96744 USA; 6 Ocean wide Science Institute, PO Box 61692, Honolulu, HI 96744 USA; 7 Society for the Study of Cetaceans in the Canary Archipelago (SECAC). Casa de Los Arroyo, Avda. Coll n°6, Apartado de Correos 49 de Arrecife de Lanzarote, 35500 Arrecife, Lanzarote Spain; 8 Sea Mammal Research Unit, Scottish Oceans Institute, University of St Andrews, St Andrews, Scotland; 9 MARE – Marine and Environmental Sciences Centre, Lisbon, Portugal; 10 Biology Department, Woods Hole Oceanographic Institution, Woods Hole, MA 02543 USA

**Keywords:** Communication signals, Acoustic divergence, Geographic variability, Phenotypic diversity, Cetaceans

## Abstract

**Background:**

Prioritizing groupings of organisms or ‘units’ below the species level is a critical issue for conservation purposes. Several techniques encompassing different time-frames, from genetics to ecological markers, have been considered to evaluate existing biological diversity at a sufficient temporal resolution to define conservation units. Given that acoustic signals are expressions of phenotypic diversity, their analysis may provide crucial information on current differentiation patterns within species. Here, we tested whether differences previously delineated within dolphin species based on i) geographic isolation, ii) genetics regardless isolation, and iii) habitat, regardless isolation and genetics, can be detected through acoustic monitoring. Recordings collected from 104 acoustic encounters of *Stenella coeruleoalba, Delphinus delphis* and *Tursiops truncatus* in the Azores, Canary Islands, the Alboran Sea and the Western Mediterranean basin between 1996 and 2012 were analyzed. The acoustic structure of communication signals was evaluated by analyzing parameters of whistles in relation to the known genetic and habitat-driven population structure.

**Results:**

Recordings from the Atlantic and Mediterranean were accurately assigned to their respective basins of origin through Discriminant Function Analysis, with a minimum 83.8% and a maximum 93.8% classification rate. A parallel pattern between divergence in acoustic features and in the genetic and ecological traits within the basins was highlighted through Random Forest analysis. Although it is not yet possible to establish a causal link between each driver and acoustic differences between basins, we showed that signal variation reflects fine-scale diversity and may be used as a proxy for recognizing discrete units.

**Conclusion:**

We recommend that acoustic analysis be included in assessments of delphinid population structure, together with genetics and ecological tracer analysis. This cost-efficient non-invasive method can be applied to uncover distinctiveness and local adaptation in other wide-ranging marine species.

**Supplementary Information:**

The online version contains supplementary material available at 10.1186/s40850-021-00085-7.

## Background

Distinguishing unambiguous groupings of organisms, or ‘units’, that are relevant to the proper implementation of management actions is still a matter of debate [1, 2]. In order to be applied to a wide range of taxa, a context-based flexible definition is required, given that different approaches may work more efficiently than others in relation to the situational circumstances [3].

A Unit of Conservation (UC) can be defined as a segment within a species to be considered distinct for conservation purposes [4]. The most prominent and discussed conservation units are Evolutionarily Significant Units (ESUs) and Management Units (MUs). The ESU criteria were first proposed by Rider [5] and subsequently reformulated to integrate information about genetic distinctiveness with data regarding adaptive variation based on ecological features [6, 7]. Management Units are considered as distinct units at a smaller scale compared to ESUs, demographically independent and important for ensuring long-term persistence of species [4]. However, genetic differences identified at the mtDNA and microsatellites levels, that have influenced the majority of the management actions carried out in the last decades, may fail in accurately defining units for conservation purposes over a short-time scale. Due to the difficulty in operationally applying the concept, several approaches have been considered to identify distinct units, such as ecological tracers and life-history parameters [2].

Recent developments include investigations into how variation in acoustic signals may be a line of evidence supporting the significance for conservation and management of different populations [8]. Variation can occur over short time scales through adaptive environmental diversification that fosters isolation and can drive phenotypic evolution [8]. Differences in the characteristics of acoustic signals can be determined by genetic factors [9–11], or support genetic differences, and therefore may be informative for reconstructing lineage histories [12]. Nevertheless, communication signals are adaptive and selection favours characteristics that enhance transmission quality under local conditions by reducing their masking and attenuation [13]. Therefore, selective pressures deriving from habitat characteristics can differentially and independently act on some individual traits of the signal [14, 15]. This is the case in a number of animal taxa such as insects [16], frogs [12, 17], songbirds [18, 19], primates [20–22], and marine mammals [23, 24]. Furthermore, social and cultural inheritance and gene-culture co-evolution have been suggested to play an important role in the evolution of species behaviour, such as vocalizations [25]. Finally, morpho-physiological constraints, such as those related to body size, are known to influence some frequency parameters [26].

Even though vocal diversity is high both among and within some animal species, vocal patterns have been used to reconstruct hypotheses of evolutionary histories: geographic distances and genetic variation among gibbon populations are strongly correlated with variation in song structure [27, 28], as well as between populations of Neotropical singing mice [29, 30]. However, the use of acoustic signals as a proxy for genetic divergence is still a matter of debate, particularly in species capable of vocal learning [31]. The process of vocal learning (the ability to modify or acquire acoustic signals through experience, imitation, cultural transmission or association to context) could influence the characteristics of acoustic signals. Evidence for vocal learning has been well documented, especially for birds [32–35] and cetaceans [36, 37]. As suggested by Brumm & Naguib [19] and Janik [38], learning enables a rapid adjustment of the signals to the acoustic properties of the local habitat, as well as cultural evolution and ontogenetic development [10]. Therefore, the contribution of genetics with the aim of distinguishing units can be difficult to delineate when groups overlap in the same areas.

In dolphins, intra-specific variation in the characteristics of tonal whistles has been described at macro- and micro-geographic scales [39–43] among others. Whistles are tonal signals used by many delphinid species for intra- and inter- specific communication. Many factors cause whistle variability. Whistle acoustic parameters vary independently under many local selective pressures [44], either ecological or cultural. Dolphins can also learn to develop context-specific acoustic structures [45] and individual-specific frequency modulation in signature whistles [46, 47]. Some frequency parameters of tonal signals are under morphological constraints and have been shown to contain the lowest amount of intra-specific variation for many species [48]. Frequency parameters of tonal signals have low variability and may be good candidates for determining divergence [49]. Nevertheless, dolphins’ whistle parameters have never been used for evaluating whether characteristics of acoustic signals can predict units of conservation (UCs).

Determining discrete units of conservation for dolphins can be an arduous task [50], given the difficulty of studying cetacean genetic population structure and the cost of analysis. However, due to the different pressure of local threats, such as fishing activity, pollution and marine traffic, on coastal and pelagic areas, it is crucial to define the borders of distinct UCs. Furthermore, since some dolphins species, notably bottlenose, striped and common dolphins, are considered Data Deficient by the International Union for the Conservation of Nature in European waters (and vulnerable or endangered at the Mediterranean level), it is critical to investigate which time-scale is suitable to consider for adequate management. Acoustics may provide an additional source of data that can help the understanding of discrepancies through space and over time of those discrete units [51]. Here, we investigate whether the characteristics of time-frequency contours can help identify units of conservation in species capable of vocal learning. We examine the patterns of variation in whistle time-frequency characteristics in three dolphin species, phylogenetically related [52] and all widespread both in the Mediterranean and in the Atlantic Oceans: bottlenose dolphins (*Tursiops truncatus*), short-beaked common dolphins (*Delphinus delphis*) and striped dolphins (*Stenella coeruleoalba*). In detail, we examine if whistles can be used to verify the possible existence of UCs among a poorly studied area by comparing groups defined according to three different criteria. Specifically, we tested:

1. the effect of geographic isolation;
2. the effect of genetics, regardless the isolation;
3. the effect of habitat, regardless isolation and genetics

## Methods

### The selected model

Principal features for each sampling area/species are summarized in Table 1.

**Table 1:** Summary of the model species considered in the study. For each species, the geographic region, location, and genetic situation has been shown

Species	GeographicRegion	Location	Genetics
Bottlenose dolphin	Atlantic Ocean	Azores Islands	Considered as a single Atlantic population [53]
		Canary Islands	
	Mediterranean Sea	Tyrrhenian Sea	Considered as Western Mediterranean, differentiated from Alboran Sea [54]
		Provencal Sea	
		Spanish waters	
		Alboran Sea	Considered as independent from the Western Mediterranean [54]
Short-beaked common dolphin	Atlantic Ocean	Azores Islands	Considered as a single Atlantic population [55]
		Canary Islands	
	Mediterranean Sea	Tyrrhenian Sea	Considered as Western Mediterranean, differentiated from Alboran Sea [56]
		Sardinian waters	
		Alboran Sea	Considered as independent from the Western Mediterranean [56]
Striped dolphin	Atlantic Ocean	Azores Islands	Considered as a single Atlantic population [57, 58]
		Canary Islands	
	Mediterranean Sea	Ligurian Sea	Considered as Western Mediterranean, differentiated from Alboran Sea [57–59]
		Tyrrhenian Sea	
		Provencal Sea	
		Balearic waters	
		Spanish waters	
		Alboran Sea	Considered as independent from the Western Mediterranean [59, 60]

#### Population structures

Bottlenose dolphins – Genetic studies have shown a limited amount of gene flow between Mediterranean and Atlantic populations of bottlenose dolphins [54, 61]. According to Natoli et al. [54], a very recent division is suggested for the boundary that divides the North Atlantic samples from the western Mediterranean Sea. Within the Atlantic, comparisons between the Azores and the Canaries have shown that these populations are genetically similar [55]. However, a habitat-driven population structure has been recently identified [61], and the presence of some resident individuals in the Azores area [62] might generate reproductive isolation between pods. Within the Mediterranean, Natoli et al. [54] examined nine microsatellite loci and mtDNA control region founding a divergence across the Mediterranean with boundaries possibly corresponding to the Almerian-Oran front and the Siculo-Tunisian front. Also, a distinction among Spanish and Tyrrhenian bottlenose dolphins, probably related to the habitat features that define patterns of movement, was identified by Moore [63].

Short-beaked common dolphins – Even if, mtDNA data suggested gene flow mediated by females from across the Gibraltar Strait [56], the Alboran population showed significant genetic differentiation compared to the Atlantic populations, that might be related to prey resources competition [64]. Within the Mediterranean, significant population differentiation between the Eastern and the Western (Alboran Sea) specimens at both nuclear and mtDNA markers [56] evolved recently, and is likely to have been reinforced by a recent bottleneck event [64]. Furthermore, Natoli et al. [64] suggested that the adaptation to different environments or the foraging strategies adopted might be driving factors for genetic differentiation in this species.

Striped dolphins – Genetic data based both on nuclear and mtDNA analyses report no sharing of haplotypes between the two ocean basins [57, 58, 65]. However, due to the low number of nuclear loci used and that mtDNA identifies only female mediated gene flow, a male mediate gene flow could still happen. No genetic data are available for the striped dolphins of the Azores and Canary islands. However, Burret et al. [58] proposed a high level of polymorphism within the Atlantic population, suggested also by the wide-ranging pattern of the species in the pelagic Northeast Atlantic [58, 66, 67]. In the Mediterranean, evidence of an intra-basin genetic structure has been found particularly between Eastern and Western populations [68]. Furthermore, within the Western Mediterranean, populations from Spain (Alboran Sea and Balearic Islands) seem to be different from the ones in Western Italy (Ligurian and Thyrrenyan Sea), possibly as a result of the dispersal behavior due to a combination of physical and ecological characteristics.

#### Environmental features

The Azores Archipelago is a Mid-Atlantic island chain located in between two current systems: the Gulf Stream that generates meanders and filaments from the western side, and the Azores Current propagating eastward and generating westward eddies [69]. Furthermore, the high-pressure system generates a wind stress gradient affecting transports as well as the turbulent ocean features. They generate a confluence zone, enriching the area with nutrients, and contributing to enhancing local productivity [69]. As well as the Canary Islands, both the archipelagos have a volcanic origin and are characterized by a high depth seafloor scattered by seamounts made up of summit plateaus and steep flanks. These last Islands rise off the north-west African coast. Distinct currents and countercurrents cross the Canary archipelago, making the region a complex system driven by local and remote forcing [70]. The most important is the Canary Current, fed by the easternmost branch of the Azores Current, and the Canary Upwelling Current that generates a near-permanent upwelling of relatively cool North Atlantic waters. Finally, the Eastern Boundary Current flows between the Canary Islands and the African boundary can be considered a large-scale flow with seasonal shift [70].

The Western Mediterranean can be subdivided into main regions: the Alboran Sea, the Algero-Balearic Basin, the Corso-Ligure-Provencal Basin and the Tyrrhenian Sea. The ecology and biogeography of these areas are shaped and characterized by drivers such as bottom morphology, water temperature, salinity, wind regimes, temporal thermoclines and currents, among others [71]. The Alboran Sea, between southern Spain and Morocco, is divided from the rest of the Western Mediterranean by the semipermanent Almería-Oran Front, formed by the convergence of two distinct water masses: the less saline Atlantic waters in the western area and the more saline Mediterranean waters to the east [72]. The general circulation of the basin is strongly influenced also by the complex physiography of the area made up of ridges, valleys and banks.

The Atlantic waters, coming from the Alboran sea, flows southward in the Algerian Basin, the largest of the Western Mediterranean, between the Balearic Chain and the Algerian margin. Here, an energetic mesoscale circulation pattern generates an intense inflow and outflow regime that has repercussions on biochemical parameters [73]. Therefore, locally and episodically high chlorophyll or primary production can modulate the biological activity of the ecosystems [73].

High levels of primary production are known to characterize the Corso-Ligure-Provencal Basin, where the spring phytoplankton bloom is mainly driven by the cyclonic circulation system. This system generates a frontal zone among the coastal and offshore waters and an upwelling of cold waters nutrient-rich with spatio-temporal interannual changes [74].

The Tyrrhenian Sea is located along the western coast of Italy, eastern of the islands of Corsica and Sardinia. Surface circulation of the water masses is dominated by the entrance of the Atlantic waters from southwest that splits into a vein directed north/northeast and another that proceeds farther eastward along the northern Sicilian coast [75]. The Sea is relatively deep and characterized by a large number of seamounts (64), that affect the productivity of offshore ecosystems and attract pelagic top predators [76].

Basing on the environmental and genetic features previously described, that can generate units isolated enough to be considered separately for management purposes, we tested:

1. the effect of geographic isolation by comparing whistles characteristics of dolphins inhabiting the Atlantic Ocean versus the Mediterranean Sea;
2. the effect of genetics, regardless the isolation by comparing whistles characteristics of dolphins inhabiting the Atlantic Ocean versus the Alboran Sea versus the Mediterranean Sea;
3. the effect of habitat, regardless isolation and genetics by comparing whistles characteristics of dolphins inhabiting the different localities sampled: Azores and Canary Islands (in the Atlantic Ocean), Alboran Sea, Ligurian Sea, Tyrrhenian Sea, Sardinian waters, Provençal Sea, Spanish waters and Balearic Sea (in the Mediterranean Sea).

### Sampling

**Fig. 1:**
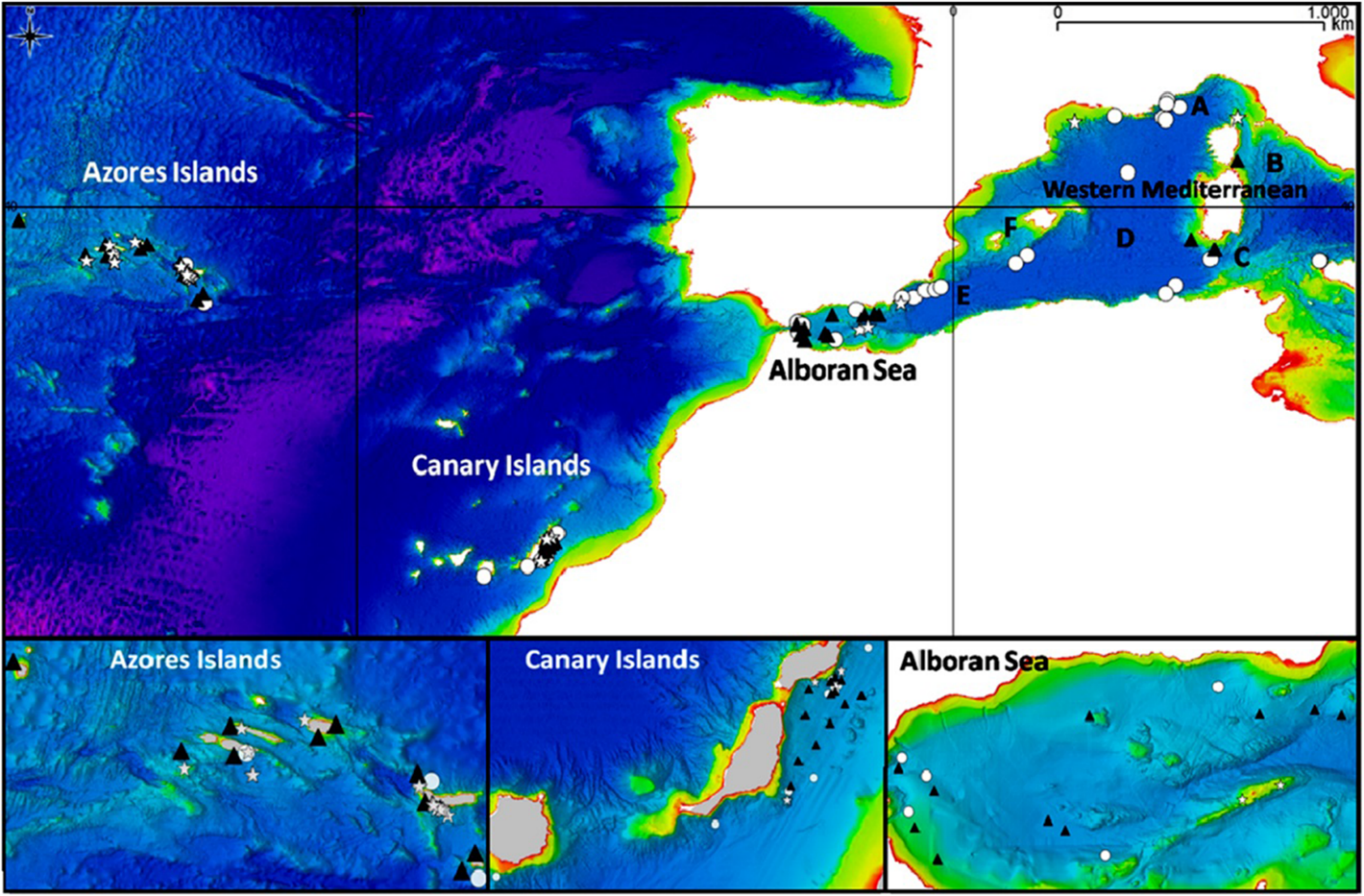
Map of the ocean basins included in the study. Two sub-areas were investigated per basin. In the Atlantic Ocean: the Azores islands and the Canary Islands. In the Mediterranean Sea: the Alboran Sea, and the Western Mediterranean (A Ligurian Sea, B Tyrrhenian Sea, C Sardinian waters, D Provençal Sea, E Spanish waters, F Balearic waters). Dots represent striped dolphin sightings, stars bottlenose dolphin sightings and triangles common dolphin sightings. Map was generated by using QGis 2.2.0 (http://qgis.org/it/site)

Dolphin groups were sampled at four locations, selected to maximize coverage of the East Atlantic and Mediterranean basins. In the Atlantic, we sampled at the Azores (between 36° and 40° latitude North and 24° and 32° longitude West), and the Canary Islands (between 27° and 30° latitude North and 13° and 19° longitude West). In the Mediterranean, we sampled groups in the Alboran Sea (between 35° and 36° latitude North and 2°and 6° longitude West), and in the Western Mediterranean (between 35° and 44° latitude North and 2° longitude West and 16° East, subdivided in the six local areas previously cited (Fig. 1).

Data were collected during vessel surveys carried out in daylight hours from 1996 to 2012. We opted for a 16-year timescale to include a wide temporal variation and to obtain more samples from distinct groups. To avoid any temporal mismatch, we reviewed published genetic and ecological information including data collected between 1990 and 2012. Species identification was visually confirmed for all acoustic recordings. Recordings collected in the presence of mixed-species groups were discarded, and we used only data collected when no groups of whistling species (other than the study-species) were present within one kilometer. The sampling effort for the three species is summarized in Table 2.

**Table 2:** Summary of the study effort for each species and each basin. For the Mediterranean, we analyzed 22.47 h of recordings from 38 sightings and we extracted 1293 whistles, 58.54% of which met good quality criteria and were analyzed. For the Atlantic Ocean, we collected 17.25 h of recordings during 63 sightings and 45.70% of 3177 signals were analyzed

	Mediterranean Sea				Atlantic Ocean			
	Hours	Sightings	Extracted Whistles	Analyzed whistles	Hours	Sightings	Extracted whistles	Analyzed whistles
**Bottlenose dolphin**	3.06	5	257	136	7.95	23	1052	420
**Short-beaked common dolphin**	8.30	14	249	120	5.60	27	984	480
**Striped dolphin**	11.11	19	787	501	3.70	13	1141	552

### Data collection

Data were collected using a variety of equipment: a mono or stereo towed Benthos hydrophone AQ4 (Teledyne Benthos North Falmouth, MA; with a flat response of 62 dB from 200 Hz to 30 kHz, a 29 dB pre-amplifier and 200 Hz high-pass filter), an HTI-94-SSQ hydrophone (High Tech Inc., Long Beach, MS; with a linear flat response of 61 dB between 1 Hz and 15 kHz, and of 63 dB between 15 and 30 kHz), an array of two Benthos AQ4, or an array of two Benthos AQ4 and two spherical ceramic hydrophone elements (Seiche Measurements Limited Bradworthy, Holsworthy, Devon, UK; with a frequency response of 2–150 kHz). Sounds were collected either on a digital tape recorder TascamVR DA-P1 (TEAC America, Inc., Montebello, CA) (with a sampling frequency of 48 kHz, 16 bit resolution, and frequency responses of 60.5 dB from 20 Hz to 20 kHz), or directly digitalized on a laptop at a sampling rate of 32 kHz, 44.1 kHz, 48 kHz or 192 kHz. Because of the differences in sampling rates, all signals collected at a sampling rate above 48 kHz were down-sampled to that value, because the maximum fundamental frequency of most whistles was found to be below 24 kHz. In recordings collected with higher sampling rates, sounds (0.81%) that went beyond the Nyquist frequency (i.e. the highest frequency that can be coded at a given sampling rate to fully reconstruct the signal, 24 kHz in this case) were not included in the analyses. Signals sampled at 32 kHz or 44.1 kHz did not contain contour sections above their Nyquist frequency.

### Acoustic data measurements

We used only whistles with sound pressure level (SPL) at least 20 dB higher than the background noise (see [42]). Nine parameters from the contour of the whistle were measured from spectrograms in CoolEdit 2.1 (Syntrillium Software, U.S.A.): signal duration, beginning, end, maximum and minimum frequency, number of inflection points (where the curvature changes sign (second derivative = 0)), steps (a discontinuous change in frequency), maxima and minima of the contour (where the slope changes sign (first derivative = 0)). These terms are described in Papale et al. [42]. After manual measurement (visual observation of the spectrogram), we checked our results by extracting the same parameters with a semi-automatic MatLab-based program (TRIA, Lammers M.O.) on a subsample of the data to prevent analyser-induced bias. There were not significant differences for any parameters except for maximum frequency (Sign test: *N* = 855, − 1.09 < Z < − 1.50, 0.13 < *P* < 0.27; for maximum freq: Z = − 8.11, *P* < 0.001). This discrepancy in the maximum frequency (mean value with the manual method = 16,678 Hz, Sd = 3623.63 Hz; mean value with the semi-automatic method = 16,411 Hz, Sd = 3342.38 Hz) is due to the low TRIA sensitivity in detecting and measuring low intensity sounds occurring at the highest frequencies. From this comparison, it emerges that human analysts can measure low intensity signals better than the automated program. To avoid pseudo-replication due to the presence of stereotyped whistles that could result from the influence of behaviour and/or social interactions, and address the independence of each whistle, sounds with similar time–frequency contours, visually matched by expert observers, were included in the analysis only once.

### Data analyses

To test the effect of geographic isolation, we performed stepwise discriminant function analyses (DFA) with cross-validation. To meet normality and homoscedasticity criteria and to reduce the weight of each whistle within a recording, we used the mean (normally distributed) values of whistle parameters recorded during each sighting (acoustic encounters) for the DFA. By using as sampling unit the acoustic encounter, and as a variable the mean of each parameter for each encounter, we also met the independence criterion. Even if social structure varies considerably among populations, dolphins living in fission–fusion societies associate in small groups that change in composition on a daily or hourly basis [77]. When in the same location, data were collected during surveys in different years and recorded at an average minimum distance of at least 10 km. If the distance was smaller than a couple of kilometers, the temporal gap between the two sightings was at least 24 h in order to prevent recording the same group of dolphins. As a consequence, the number of acoustic encounters per species ranged from 5 to 19 for the Mediterranean and 13–24 for the Atlantic Ocean. Given that small sample sizes can reduce power and affect statistical inference reliability, we considered the results reliable if the resulting discriminant model was based on only few variables, following the principle that a higher number of variables compared to the number of cases can lead to a poor discriminant rate.

To test the effect of genetics, regardless the isolation and the effect of habitat, regardless isolation and genetics, we considered the single signals as units in order to maintain all the intra-sighting variability. Due to the visual acoustic preliminary analysis previously described, potential issues of pseudo-replication were avoided. Furthermore, only sightings with at least three good quality whistles were included [see 43 for details].

In order to deal with a dataset that violates the a priori assumption of normality and homoscedasticity, we used the Random Forest (RF) machine learning method [78]. This methodological approach has been successfully implemented on structurally similar data in recent ecological studies [79–82], in which the high heterogeneity of the ecological data encourages the use of approaches not based on a priori assumptions regarding the distribution of input data.

We implemented six RF models with three species (bottlenose dolphin, short-beaked common dolphin, striped dolphin) and two response variables (genetic units, geographical groups).

We used whistle frequency (maximum, minimum, beginning and final frequency [Hz]) and duration [s] parameters in the RF models because these variables had lower Coefficients of Variation (CV) than those that describe modulation patterns (number of inflection points, of steps, of minima and of maxima). We constructed classification trees using a bootstrap aggregating algorithm [83] that allowed a reduced variance of predicted values and decreased risk of overfitting. Consequently, each tree was built on a randomly sub-sampled training dataset, while the subsequent predictions were carried out considering the remaining data (called Out-Of-Bag, OOB) allowing an unbiased estimate of the classification error. Predictor variables were selected from a random subsample of variables at each split [78]. Optimal model parameters (i.e. number of trees and number of random variables considered at each split) were identified by setting up a grid of tuning parameters to maximize correct predictions, using the OOB (Out-Of-Bag) estimate of misclassification rates as a measure of model performance. Consequently, 2500 trees and 2 random variables at each split emerged as a good compromise between optimized performance and computation time and thus were considered in the models. Variable importance was used as a measure of the contribution of each predictor variable to the fitted model. Variable importance was calculated based on the mean decrease in accuracy (MDA) for each variable, where MDA is the normalized difference of the classification accuracy between two models, one considering the original predictor and one considering a randomly permuted predictor [84]. In RF, as in all machine learning models, the class imbalance leads to inaccurate results, especially for the minority classes that could be not well predicted as poorly represented during the learning process. As the number of the sightings and the number of the whistles were not unequal among groups (Supplementary 1), we used the Synthetic Minority Over-sampling Technique (SMOTE) [85] to balance the number of observations among classes before performing RF analysis. This method carry out both an oversampling of the minority classes and an undersampling of the majority classes and is particularly appropriate as pre-processing method with the aim at equalizing the number of observation among groups before implementing machine learning techniques [86].

Random Forest models and SMOTE technique has been implemented using respectively the “randomForest” [84] and “UBL” [87] packages in R environment (v. 3.6.2), while the statistical software package PASW STATISTICS 18.0 (SPSS Institute Inc., Chicago, IL) was used for descriptive statistics of variation (mean, standard deviation, coefficients of variation).

## Results

We collected 39.72 h of recordings during 104 acoustic encounters and analyzed 2209 whistles. For the Mediterranean, we analyzed 22.47 h of recordings from 38 sightings and we extracted 1293 whistles (855 in the Western Mediterranean and 438 in the Alboran Sea, 58.54% of which met good quality criteria and were analyzed. For the Atlantic Ocean, we collected 17.25 h of recordings during 63 sightings and 45.70% of 3177 (1516 whistles collected in the Azores and 1661 collected in the Canary Islands) signals were analyzed. Data collection is summarized in Table 2.

All whistle parameters exhibited intra-specific variation higher than 10%. By contrasting variability of frequency parameters with those of duration and modulation (number of inflection points, of steps, of minima and of maxima), we found that CVs of signal duration were intermediate (ranging from 27.19 to 66.97%) between those of frequency (ranging from 10.19 to 46.21%) and modulation parameters (ranging from 65.12 to 230.44%).

### The effect of geographic isolation

The values of parameters obtained from Mediterranean and Atlantic whistles allowed us to correctly assign, through DFA, more than 83% of whistles to their basin of origin: 93.8% for striped dolphin, 85.7% for bottlenose dolphin and 83.8% for short-beaked common dolphin (respectively: Fisher’s F = 21.10, Wilks’ Lambda = 0.41,*P* < 0.001, coefficients of the function: number of inflection points = − 1.40, number of minima = 1.46; Fisher’s F = 28.02, Wilks’ Lambda = 0.54, *P* < 0.001, coefficients of the function: end frequency = 0.90, number of inflection points = 0.82 Fisher’s F = 10.73, Wilks’ Lambda = 0.38, *P* < 0.001, coefficients of the function: number of inflection points = 1.42, number of maxima = − 0.94) (Table 3). The number of inflection points contributed to the distinction for all three species, while the number of maxima contributed only for common dolphin, the number of minima for striped dolphin, and the end frequency only for bottlenose dolphin. Correct assignment of the smallest sample was within the range of all the assignments obtained in the DFA (Mediterranean bottlenose dolphin 80%).

**Table 3:** Detailed results of the DFAs (all significant *P* < 0.001) obtained from Mediterranean and Atlantic recordings. All whistles parameters were used to built the model. The range of the correct assignment is 66.7–100% for the Mediterranean Sea and 84.6–92.0% for the Atlantic Ocean

			Predicted group membership (%)	
Bottlenose dolphin			Mediterranean Sea	Atlantic Ocean
	Original	Mediterranean Sea (*n* = 5)	80.0	20.0
		Atlantic Ocean (*n* = 23)	13.0	87.0
	Cross-validated	Mediterranean Sea	80.0	20.0
		Atlantic Ocean	13.0	87.0
Short-beaked common dolphin				
	Mediterranean Sea	Atlantic Ocean		
	Original	Mediterranean Sea(*n* = 14)	66.7	33.3
		Atlantic Ocean (*n* = 27)	8.0	92.0
	Cross-validated	Mediterranean Sea	66.7	33.3
		Atlantic Ocean	8.0	92.0
Striped dolphin			Mediterranean Sea	Atlantic Ocean
	Original	Mediterranean Sea (*n* = 19)	100.0	0.0
		Atlantic Ocean (*n* = 13)	7.7	92.3
	Cross-validated	Mediterranean Sea	100.0	0.0
		Atlantic Ocean	15.4	84.6

### The effect of genetics, regardless the isolation

**Fig. 2:**
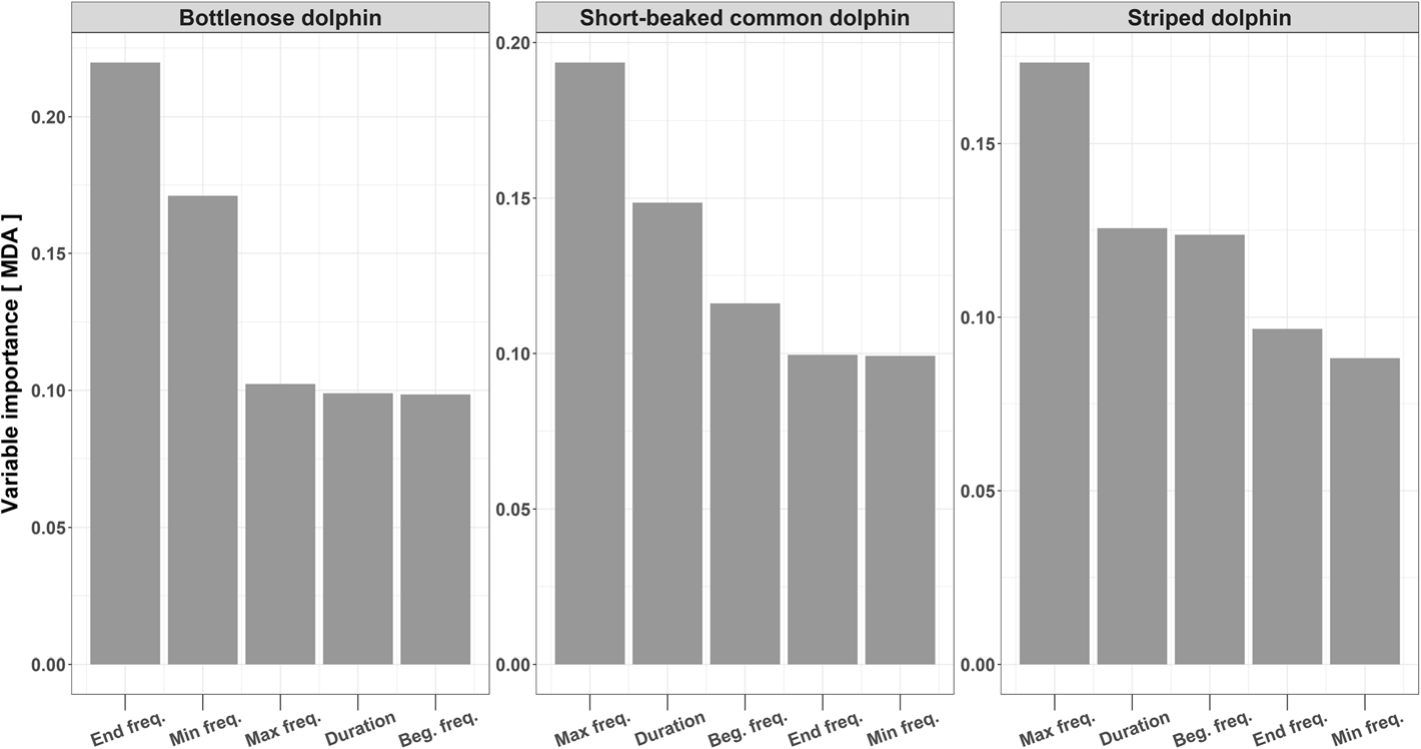
Variable importance for the correct classifications of the groups as estimated by the Random Forest Analysis performed in order to determine the effect of genetics on whistle structure regardless isolation (i.e. using samples from the Mediterranean Sea, the Alboran Sea and the Atlantic Ocean)

Given the strong match between genetic and acoustic divergence recorded for the individuals living in the Atlantic Ocean and in the Mediterranean Sea, we examined whether a similar pattern could be found when considering the groups of the western Mediterranean Sea, the Alboran Sea and the Atlantic Ocean. Random forest analysis resulted in low estimated values of classification error (OOB estimate of error rate: bottlenose dolphin = 16.61%; short-beaked common dolphin = 22.69%; striped dolphin = 25.97%), showing a high level of discrimination among the genetic groups considered. Variable importance analysis allowed us to identify the variables that contributed the most to classification accuracy (Fig. 2). End and minimum frequencies exerted the most influence in discriminating bottlenose dolphins among the three areas, while maximum frequency, duration of the signals and beginning frequency were the most relevant parameters for distinguishing short beaked common and striped dolphins among the three areas (Fig. 2).

### The effect of habitat, regardless isolation and genetics

Given that many factors act on acoustic parameters, we verified to what extent the general scenario was distinguishable, both within the Mediterranean and the Atlantic. Tests performed in relation to local areas showed that, for all the species, the OOB estimate of the classification error was in any case lower than 38%, in spite of the higher number of groups considered, which ranged between five (short-beaked common dolphin) and eight (striped dolphin) (Supplementary 1). Within the Atlantic, end and minimum frequency continued to have the highest importance for correct classification of bottlenose dolphin observations. In the other two species, maximum frequency remained the most influential parameter. Within the Mediterranean Sea, high discrimination was highlighted for all the species. For bottlenose dolphins, minimum frequency remained important for distinguishing among groups; duration remained the most important parameter for distinguishing short-beaked common dolphins groups, while maximum frequency remained the most important parameter for striped dolphins. In all three cases, the beginning frequency was also an important parameter in the classification models.

## Discussion

Our results suggest that the features of delphinids’ whistles may be useful to outline the presence of distinct groups basing on genetic and environmental features. Examining the effect of geographic isolation, the effect of genetics, regardless the isolation and the effect of habitat, regardless isolation and genetics on the vocalizations of the three different species studied, we provide a new framework for characterize differentiation and inform management decisions.

Distinct UCs, isolated by the geographic barrier created by the Strait of Gibraltar (Atlantic vs. Mediterranean) can be identified acoustically at a correct classification score to greater than 83%. Differences in the spectral and temporal features of whistles matched the geographic isolation suggesting that it may translate into differences in acoustic parameters. Samarra et al. [88] found a similar result by analyzing killer whale’s whistles recorded in the Atlantic and in the Pacific Ocean. Indeed, isolated killer whales’ populations showed a stronger divergence in frequency parameters compared to the variation detected at an intra-basin level (i.e. Iceland and Norway). However, genetically distinct killer whales, not completely geographically isolated, exhibited a level of variation still useful for distinguishing populations. In agreement with this study, the variability of dolphin whistles observed are consistent with genetic differences, also regardless geographic isolation and reflects population structure. As suggested by Samarra et al. [88], acoustic differences may reflect both historical geographic isolation and a more recent divergence between adjacent populations.

The discriminant power of acoustic signals is prominent between ocean basins, where the gene flow is presumably lower than it is within ocean basins. However, acoustic analysis reveals to be once again a good tool to potentially delineate the range of different genetic groups even over geographically close areas.

A relation among genetic features and acoustic pattern has been already demonstrated also for North Atlantic fin whales [89] and sperm whales [90]. In these cases, acoustic variability has been linked to segregation possibly generated by dispersal range and/or social characteristics. The causes of variation can be different among species, since the variability might partially derive from other factors which can generate local changes in the acoustic characteristics of signals, independently of genetic or ecological differences, such as group size, group composition, behavioural state and vocal learning [44, 91]. Animal culture and social structure indeed have the potential to affect acoustic processes in several ways [92]. As said before, delphinid species are capable of both vertical (from parents to offspring) and horizontal (among peers) cultural transmission [37]. Their social structure and cultural changes could play a crucial role in driving isolation among pods, and promoting different reactions to local conditions, highlighting the role of gene–culture coevolution in acoustic processes. In this work, we did not consider these parameters that, due to the sampling design, could be over-represented or not represented, and therefore could possibly have an influence on the results.

However, our results show also that whistles can be predictive of the finer-scale habitat-driven population structure, both in the Atlantic and in the Mediterranean, where dolphin populations are known to be structured based on local habitat dependencies [54, 56, 59, 61, 68]. Habitat features (both environmental and anthropogenic) are considered drivers of whistle changes [24, 44, 93] and their variation could represent an adaptation to signal transmission in the environment or caused by genetic differences related to the habitat niche differentiation. Recently, genetic differentiations have been detected in the form of offshore and coastal ecotypes, in particular for *Tursiops truncatus* both in the Atlantic and in the Mediterranean [63]. However, since information is still scarce in some areas for the species considered, and our samples were obtained both in coastal and in offshore waters, different ecotypes could have been sampled. Therefore, even though the Random Forest classification model highlighted that the whistles were highly classified to the assigned group, the current variability should be better investigated. Indeed, our limited sample size may not capture all of the variability in the whistle repertoires of these populations and may not provide a complete picture of the similarities and differences between populations. Considering a higher number of recordings and increasing the sampling area could reveal possible connections or stronger differences among groups.

## Conclusions

The preliminary map of acoustic patterns drawn from this study suggests that comparison of the acoustic characteristics of whistles can be a tool to complement genetic methods usually applied to identify distinct UCs for at least some delphinid species.

We recommend that acoustic analysis be embedded in assessments of delphinid population structure, together with genetics and ecological tracer analysis. Passive acoustic monitoring systems represent a cost-efficient non-invasive method to collect signals for identifying potential units of conservation, based on its correlation with other lines of evidence (e.g. genetic data). Acoustics studies provide a framework to guide population viability analyses that can be applied over larger spatial and temporal scale, improving efforts in the management of units in need of conservation.

## Supplementary Information

**Additional file 1.** Supplementary 1 Results of the Random Forest Analysis. Mediterranean and Atlantic sites are indicated by the letters M and A respectively. Acoustic parameters are reported in order of variable importance as estimated by the model and the most important variables (i.e. Mean Decrease Accuracy > 0.2) are indicated in bold. Names of acoustic parameters are abbreviated as follows: dur = duration; beg.f = beginnig frequency; end.f = end frequency; min.f = minimum frequency; max.f = maximum frequency.

## Data Availability

The datasets generated during the current study are available from the corresponding author on reasonable request.

## Abbreviations

UC: Unit of Conservation
ESU: Evolutionarily Significant Unit
MU: Management Unit
mtDNA: mitochondrial DNA
SPL: Sound Pressure Level
DFA: Discriminant Function Analysis
RF: Random Forest
OOB: Out-of-Bag
MDA: mean decrease in accuracy
SMOTE: Synthetic Minority Over-sampling Technique
