# Supplementary material for: Dolphin whistles can be useful tools in identifying units of conservation
Source: BMC Zool. 2021 Jul 29;6:22. doi: 10.1186/s40850-021-00085-7 (PMC10127015; doi:10.1186/s40850-021-00085-7)
Supplement: Supplementary file 1 — Additional file 1. Supplementary 1 Results of the Random Forest Analysis. Mediterranean and Atlantic sites are indicated by the letters M and A respectively. Acoustic parameters are reported in order of variable importance as estimated by the model and the most important variables (i.e. Mean Decrease Accuracy > 0.2) are indicated in bold. Names of acoustic parameters are abbreviated as follows: dur = duration; beg.f = beginnig frequency; end.f = end frequency; min.f = minimum frequency; max.f = maximum frequency. [file 40850_2021_85_MOESM1_ESM.docx]

**Dolphin whistles contribute to identifying Evolutionary Significant Units**

Papale Elena^ab^, Azzolin Marta^b^, Cascão Irma^c^, Gannier Alexandre^d^, Lammers Marc Olav^e^, Martin Vidal Martel^f^, Oswald Julie Nicola^g^, Perez-Gil Monica^f^, Prieto Rui^ch^, Silva Mónica Almeida^ci^, Torri Marco^a^, Giacoma Cristina^b^

Corresponding author: ^a^Institutefor Anthropogenic Impacts and Sustainability in the Marine Environment (CNR-IAS), detached unitof Capo Granitola, National Research Council, Via del Mare 3, 91021 Campobello di Mazara (TP), Italy. E-mail: elena.papale@cnr.it

^a^Institutefor Anthropogenic Impacts and Sustainability in the Marine Environment (CNR-IAS), detached unitof Capo Granitola, National Research Council, Via del Mare 3, 91021 Campobello di Mazara (TP), Italy.

^b^ University of Torino, Life Sciences and Systems Biology Department, Via Accademia Albertina 13, 10123 Torino, Italy.

^c^ IMAR – Institute of Marine Research & OKEANOS R&D Centre; University of the Azores, Horta, Portugal

^d^Groupe de Recherche sur les Cétacés, Antibes, France.

^e^Hawaii Institute of Marine Biology, University of Hawaii, Kaneohe, HI 96744, USA and Ocean wide Science Institute, PO Box 61692, Honolulu, HI 96744, USA.

^f^ Society for the Study of Cetaceans in the Canary Archipelago (SECAC). Casa de Los Arroyo, Avda. Coll nº6, 35500 Arrecife. Lanzarote, Spain. Apartado de Correos 49 de Arrecife de Lanzarote.

^g^ Sea Mammal Research Unit, Scottish Oceans Institute, University of St Andrews, St Andrews, Scotland.

^h^MARE – Marine and Environmental Sciences Centre, Lisbon, Portugal

^I^ Biology Department, Woods Hole Oceanographic Institution, Woods Hole, MA 02543, USA.

Supplementary 1 Results of the Random Forest Analysis. Mediterranean and Atlantic sites are indicated by the letters M and A respectively. Acoustic parameters are reported in order of variable importance as estimated by the model and the most important variables (i.e. Mean Decrease Accuracy >0.2) are indicated in bold. Names of acoustic parameters are abbreviated as follows: dur = duration; beg.f = beginnig frequency; end.f = end frequency; min.f = minimum frequency; max.f = maximum frequency.

| **Bottlenose dolphins** | Original sample size | Balanced sample size |  | Classification Error | Western Mediterranean | Alboran Sea | Atlantic Ocean |  |  |  |  |  |  | Variable importance for the correct classification of the single groups |
| --- | --- | --- | --- | --- | --- | --- | --- | --- | --- | --- | --- | --- | --- | --- |
| OOB estimate of error rate = 16.61% Overall variable importance: **end.f;**min.f; max.f; beg.f; dur | **n=24** | **n=185** | Western Mediterranean | 0.06 | **0.94** | 0.03 | 0.03 |  |  |  |  |  |  | **end.f; min.f; max.f**; dur; beg.f |
|  | **n=112** | **n=185** | Alboran Sea | 0.2 | 0.05 | **0.8** | 0.15 |  |  |  |  |  |  | end.f; beg.f; min.f; dur; max.f |
|  | **n=420** | **n=185** | Atlantic Ocean | 0.24 | 0.05 | 0.19 | **0.76** |  |  |  |  |  |  | end.f; min.f; max.f; beg.f; dur |
|  |  |  |  |  |  |  |  |  |  |  |  |  |  |  |
|  |  |  |  |  | Ligurian Sea (M) | Provencal Sea (M) | Tyrrhenian Sea (M) | Sardinian waters (M) | Spanish waters (M) | Balearic Islands (M) | Alboran Sea (M) | Azores Islands (A) | Canary Islands (A) |  |
| OOB estimate of error rate = 26.34% Overall variable importance: **beg.f; min.f; end.f;**dur, max.f | - | - | Ligurian Sea (M) | - | - | - | - | - | - | - | - | - | - |  |
|  | **n=7** | **n=93** | Provencal Sea (M) | 0.04 | - | **0.96** | 0.04 | - | 0 | - | 0 | 0 | 0 | end.f; beg.f; dur; min.f; max.f |
|  | **n=9** | **n=93** | Tyrrhenian Sea (M) | 0.11 | - | 0.05 | **0.89** | - | 0 | - | 0.04 | 0.01 | 0 | **min.f; end.f; beg.f**; dur; max.f |
|  | - | - | Sardinian waters (M) | - | - | - | - | - | - | - | - | - | - |  |
|  | **n=8** | **n=93** | Spanish waters (M) | 0.05 | - | 0 | 0.01 | - | **0.95** | - | 0 | 0.02 | 0.02 | **beg.f; min.f; end.f**; dur; max.f |
|  | - | - | Balearic Islands (M) | - | - | - | - | - | - | - | - | - | - |  |
|  | **n=112** | **n=93** | Alboran Sea (M) | 0.44 | - | 0.04 | 0.09 | - | 0 | - | **0.56** | 0.21 | 0.1 | end.f; min.f; beg.f; dur; max.f |
|  | **n=326** | **n=93** | Azores Islands (A) | 0.56 | - | 0.01 | 0.06 | - | 0.01 | - | 0.27 | **0.44** | 0.21 | min.f; end.f; dur; beg.f; max.f; |
|  | **n=94** | **n=93** | Canary Islands (A) | 0.38 | - | 0.02 | 0.02 | - | 0.02 | - | 0.12 | 0.19 | **0.63** | max.f; end.f; beg.f; dur; min.f |
|  |  |  |  |  |  |  |  |  |  |  |  |  |  |  |
|  |  |  |  |  |  |  |  |  |  |  |  |  |  |  |
| **Short-beaked common dolphins** |  |  |  |  | Western Mediterranean | Alboran Sea | Atlantic Ocean |  |  |  |  |  |  |  |
| OOB estimate of error rate = 22.86% Overall variable importance: **max.f;**dur; beg.f; end.f; min.f | **n=37** | **n=198** | Western Mediterranean | 0.1 | **0.9** | 0.05 | 0.05 |  |  |  |  |  |  | **max.f; dur;**beg.f; end.f; min.f |
|  | **n=80** | **n=198** | Alboran Sea | 0.21 | 0.05 | **0.79** | 0.16 |  |  |  |  |  |  | max.f; dur; min.f; beg.f; end.f |
|  | **n=480** | **n=198** | Atlantic Ocean | 0.37 | 0.13 | 0.24 | **0.63** |  |  |  |  |  |  | max.f; dur; beg.f; end.f; min.f |
|  |  |  |  |  |  |  |  |  |  |  |  |  |  |  |
|  |  |  |  |  | Ligurian Sea (M) | Provencal Sea (M) | Tyrrhenian Sea (M) | Sardinian waters (M) | Spanish waters (M) | Balearic Islands (M) | Alboran Sea (M) | Azores Islands (A) | Canary Islands (A) |  |
| OOB estimate of error rate = 34.12% Overall variable importance: **dur**;**beg.f**; **max.f**; min.f; end.f | - | - | Ligurian Sea (M) | - | - | - | - | - | - | - | - | - | - |  |
|  | - | - | Provencal Sea (M) | - | - | - | - | - | - | - | - | - | - |  |
|  | **n=29** | **n=119** | Tyrrhenian Sea (M) | 0.09 | - | - | **0.91** | 0.01 | - | - | 0.02 | 0.04 | 0.02 | **max.f; dur;**end.f; beg.f; min.f |
|  | **n=8** | **n=119** | Sardinian waters (M) | 0.06 | - | - | 0.01 | **0.95** | - | - | 0 | 0.02 | 0.02 | **beg.f;min.f**; **end.f**; **max.f**; **dur** |
|  | - | - | Spanish waters (M) | - | - | - | - | - | - | - | - | - | - |  |
|  | - | - | Balearic Islands (M) | - | - | - | - | - | - | - | - | - | - |  |
|  | **n=80** | **n=119** | Alboran Sea (M) | 0.38 | - | - | 0.02 | 0.02 | - | - | **0.63** | 0.18 | 0.15 | min.f; max.f; dur; end.f; beg.f |
|  | **n=191** | **n=119** | Azores Islands (A) | 0.51 | - | - | 0.03 | 0.07 | - | - | 0.23 | **0.49** | 0.18 | max.f; beg.f; dur; end.f; min.f |
|  | **n=289** | **n=119** | Canary Islands (A) | 0.66 | - | - | 0.18 | 0.05 | - | - | 0.19 | 0.24 | **0.34** | dur; beg.f; max.f; min.f; end.f |
|  |  |  |  |  |  |  |  |  |  |  |  |  |  |  |
|  |  |  |  |  |  |  |  |  |  |  |  |  |  |  |
| **Striped dolphins** |  |  |  |  | Western Mediterranean | Alboran Sea | Atlantic Ocean |  |  |  |  |  |  |  |
| OOB estimate of error rate = 27.17% Overall variable importance: **max.f;**dur; beg.f; end.f; min.f | **n=501** | **n=361** | Western Mediterranean | 0.37 | **0.63** | 0.07 | 0.3 |  |  |  |  |  |  | max.f; dur; beg.f; end.f; min.f |
|  | **n=31** | **n=361** | Alboran Sea | 0.06 | 0.04 | **0.94** | 0.02 |  |  |  |  |  |  | **max.f; dur; min.f; beg.f; end.f** |
|  | **n=552** | **n=361** | Atlantic Ocean | 0.39 | 0.29 | 0.09 | **0.61** |  |  |  |  |  |  | max.f; dur; end.f; beg.f; min.f |
|  |  |  |  |  |  |  |  |  |  |  |  |  |  |  |
|  |  |  |  |  | Ligurian Sea (M) | Provencal Sea (M) | Tyrrhenian Sea (M) | Sardinian waters (M) | Spanish waters (M) | Balearic Islands (M) | Alboran Sea (M) | Azores Islands (A) | Canary Islands (A) |  |
| OOB estimate of error rate = 37.79% Overall variable importance: **max.f**; **beg.f**; **min.f**;dur; end.f | **n=204** | **n=135** | Ligurian Sea (M) | 0.54 | **0.47** | 0.11 | 0.04 | - | 0.15 | 0.07 | 0.04 | 0.05 | 0.07 | max.f; dur; min.f; beg.f; end.f |
|  | **n=60** | **n=135** | Provencal Sea (M) | 0.29 | 0.11 | **0.71** | 0.01 | - | 0.07 | 0.02 | 0.02 | 0.02 | 0.04 | **min.f; max.f;**beg.f; dur; end.f |
|  | **n=35** | **n=135** | Tyrrhenian Sea (M) | 0.09 | 0.02 | 0.01 | **0.92** | - | 0 | 0.02 | 0.01 | 0 | 0.02 | **max.f; dur; min.f; end.f;**beg.f |
|  | - | - | Sardinian waters (M) | - | - | - | - | - | - | - | - | - | - |  |
|  | **n=138** | **n=135** | Spanish waters (M) | 0.62 | 0.17 | 0.13 | 0.06 | - | **0.38** | 0.06 | 0.07 | 0.07 | 0.06 | max.f; beg.f; min.f; dur; end.f |
|  | **n=52** | **n=135** | Balearic Islands (M) | 0.21 | 0.08 | 0.05 | 0.01 | - | 0.03 | **0.79** | 0.02 | 0.01 | 0.01 | **beg.f; min.f;**max.f; dur; end.f |
|  | **n=43** | **n=135** | Alboran Sea (M) | 0.22 | 0.01 | 0.01 | 0.01 | - | 0.05 | 0.02 | **0.78** | 0.07 | 0.05 | **max.f; min.f; beg.f; dur;**end.f |
|  | **n=110** | **n=135** | Azores Islands (A) | 0.44 | 0.07 | 0.06 | 0.05 | - | 0.06 | 0.05 | 0.08 | **0.56** | 0.07 | max.f; end.f; min.f; beg.f; dur |
|  | **n=442** | **n=135** | Canary Islands (A) | 0.61 | 0.12 | 0.05 | 0.07 | - | 0.15 | 0.05 | 0.09 | 0.08 | **0.39** | dur; beg.f; min.f; max.f; end.f |
